# Role of phosphatidylserine in the localization of cell surface membrane proteins in yeast

**DOI:** 10.1247/csf.22081

**Published:** 2022-12-15

**Authors:** Ryutaro Kashikuma, Makoto Nagano, Hiroki Shimamura, Kouya Nukaga, Ikumi Katsumata, Junko Y. Toshima, Jiro Toshima

**Affiliations:** 1 Department of Biological Science and Technology, Tokyo University of Science, 6-3-1 Niijyuku, Katsushika-ku, Tokyo 125-8585, Japan; 2 School of Health Science, Tokyo University of Technology, 5-23-22 Nishikamata, Ota-ku, Tokyo 144-8535, Japan

**Keywords:** phosphatidylserine, endocytosis, recycling, vesicle transport

## Abstract

Phosphatidylserine (PS) is a constituent of the cell membrane, being especially abundant in the cytoplasmic leaflet, and plays important roles in a number of cellular functions, including the formation of cell polarity and intracellular vesicle transport. Several studies in mammalian cells have suggested the role of PS in retrograde membrane traffic through endosomes, but in yeast, where PS is localized primarily at the plasma membrane (PM), the role in intracellular organelles remains unclear. Additionally, it is reported that polarized endocytic site formation is defective in PS-depleted yeast cells, but the role in the endocytic machinery has not been well understood. In this study, to clarify the role of PS in the endocytic pathway, we analyzed the effect of PS depletion on endocytic internalization and post-endocytic transport. We demonstrated that in cell lacking the PS synthase Cho1p (*cho1*Δ cell), binding and internalization of mating pheromone α-factor into the cell was severely impaired. Interestingly, the processes of endocytosis were mostly unaffected, but protein transport from the *trans*-Golgi network (TGN) to the PM was defective and localization of cell surface proteins was severely impaired in *cho1*Δ cells. We also showed that PS accumulated in intracellular compartments in cells lacking Rcy1p and Vps52p, both of which are implicated in endosome-to-PM transport via the TGN, and that the number of Snx4p-residing endosomes was increased in *cho1*Δ cells. These results suggest that PS plays a crucial role in the transport and localization of cell surface membrane proteins.

## Introduction

Phosphatidylserine (PS) is an anionic phospholipid present in the eukaryotic cell membrane, where it is a major constituent of the cytosolic leaflet. It is known to play important roles in various biological processes, including blood coagulation, phagocytosis of apoptotic cells, and recruitment of signaling molecules (Fadok *et al.*, 1992; Lentz, 2003; Lucas and Cho, 2011). Additionally, PS dysregulation is known to be associated with different neurological diseases, such as Alzheimer’s disease and Parkinson’s disease (Ma *et al.*, 2022). Certain viruses, such as Human Immunodeficiency Virus and Dengue Virus, are known to invade host cells through binding to PS in the outer leaflet of the plasma membrane (PM) (Moller-Tank and Maury, 2014). Therefore, to develop potential therapeutic targets for these diseases, it is important to clarify the cellular processes regulated by PS.

Previous studies have reported that PS is polarized at the PM and required for the development of cell polarity (Fairn *et al.*, 2011). The highly conserved small GTPase, Cdc42, regulates the formation of cell polarity in eukaryotic cells (Etienne-Manneville, 2004; Pichaud *et al.*, 2019). The yeast *Saccharomyces cerevisiae* forms polarized projections toward mating partners, bringing the cells into direct contact (Barral *et al.*, 2000). Binding of the mating pheromone α-factor to the receptor Ste2p leads to recruitment of Cdc24, a guanine nucleotide exchange factor (GEF) for Cdc42p, and promotes Cdc42p activation at the tip of the mating projections, causing actin polymerization at presumptive bud sites (Butty *et al.*, 2002; Wiget *et al.*, 2004). In contrast, yeast mutants lacking the PS synthase, Cho1p (*cho1*Δ), show impairment of polarized Cdc42p localization, leading to a delay in bud emergence and defective mating (Fairn *et al.*, 2011). Since the secretory pathway is required for the polarized distribution of PS (Fairn *et al.*, 2011), intracellular vesicle transport seems to be important for the establishment of cell polarity, but the roles of PS in vesicle transport remain insufficiently understood. Additionally, it has not been clarified why PS depletion causes defective formation of mating projections.

Over the last decade, several probes, such as the C2 domain of lactadherin (Lact-C2) and the PH domain of evectin-2 (evt-2 PH), that bind to PS with high affinity have been developed, and studies using these probes have revealed that PS localizes to the PM and intracellular compartments in the endocytic pathway (Moravcevic *et al.*, 2010; Uchida *et al.*, 2011; Yeung *et al.*, 2008). Sun and Drubin have reported the function of two anionic phospholipids, PI(4,5)P2 and PS, during clathrin-mediated endocytosis site initiation and vesicle formation in yeast (Sun and Drubin, 2012). They demonstrated that PI(4,5)P2 is essential for endocytic membrane invagination, whereas PS is important for the efficient recruitment and spatial restriction of endocytic proteins, such as the late clathrin coat protein Sla1p, to the PM (Sun and Drubin, 2012). Several studies in mammalian cells have revealed the role of PS in retrograde membrane traffic through endosomes (Kawasaki *et al.*, 2022; Lee *et al.*, 2015; Uchida *et al.*, 2011). Uchida *et al.* have demonstrated that PS is highly concentrated in recycling endosomes (REs), thereby targeting evectin-2 to REs via its PH domain and controlling RE-to-Golgi transport (Uchida *et al.*, 2011).

EHD1 (Eps15 homology domain-containing protein 1), a dynamin-like ATPase functioning in the endocytic pathway, is also shown to localize at endosomes by binding to PS (Lee *et al.*, 2015; Naslavsky and Caplan, 2011). EHD1 is implicated in the retrograde transport mediated by SNX-BAR proteins, and knockdown of EHD1 causes defective recycling of endocytosed transferrin from REs to the PM (Lee *et al.*, 2015; McKenzie *et al.*, 2012). These observations suggest a specific role of PS at the endosomal compartment in mammalian cells. In yeast, PS was shown to distribute in various organelles, including the PM, Golgi, and vacuole (Tsuji *et al.*, 2019), but it is still unclear whether PS has a specific role in endosomal compartments. Several studies have shown that type 4 P-type ATPases, which translocate PS from the extracellular to cytoplasmic leaflet of the cell membrane, are involved in endosome-to-Golgi transport (Furuta *et al.*, 2007; Sakane *et al.*, 2006), suggesting a role of PS in intracellular vesicle transport.

In the present study we show that in PS-deficient *cho1*Δ cells, binding and internalization of mating pheromone α-factor into the cell is impaired due to the decreased localization of receptor at the PM. We further demonstrate that localization of cell surface proteins at the PM is impaired in *cho1*Δ cells, and that these proteins are mis-sorted to the vacuole. We have also demonstrated that PS depletion causes an increase in the number of Snx4-residing endosomes. These findings suggest that PS plays a crucial role in the transport and localization of cell surface proteins to the PM.

## Material and Methods

### Yeast strains

The yeast strains used in this study are listed in Table S1. All strains were grown at 25°C in standard rich medium (YPD) or synthetic medium (SM) supplemented with 2% glucose and appropriate amino acids. The C-terminal fluorescent protein tagging of proteins was performed as described previously (Toshima *et al.*, 2014)

### Fluorescence microscopy and image analysis

Fluorescence microscopy was performed using an Olympus IX83 microscope equipped with a ×100/NA 1.40 (Olympus) or a ×100/NA 1.49 (Olympus) objective and Orca-R2 cooled CCD camera (Hamamatsu), using Metamorph software (Universal Imaging). Double-color imaging were performed using an Olympus IX81 microscope equipped with a high-speed filter changer (Lambda 10-3; Sutter Instruments) that can change filter sets within 40 ms. Simultaneous imaging of red and green fluorescence was performed using an Olympus IX83 microscope, described above, and an image splitter (Dual-View; Optical Insights) that divided the red and green components of the images with a 565-nm dichroic mirror and passed the red component through a 630/50 nm filter and the green component through a 530/30 nm filter. These split signals were taken simultaneously with one CCD camera, described above. All cells were imaged during the early- to mid-logarithmic phase. Images for analysis of co-localization of red and green signals were acquired using simultaneous imaging (64.5 nm pixel size), described above.

### Fluorescence labeling of α-factor and endocytosis assays

Fluorescence labeling of α-factor was performed as described previously (Toshima *et al.*, 2006). For endocytosis assays, cells were grown to an OD_600_ of ~0.5 in 0.5 ml YPD, briefly centrifuged, and resuspended in 20 μl SM with 5 μM Alexa Fluor 594-α-factor. After incubation on ice for 2 h, the cells were washed with ice-cold SM. Internalization was initiated by the addition of SM containing 4% glucose and amino acids at 25°C.

### ^35^S-labeled α-factor internalization and binding assays

Preparation and internalization of ^35^S-labeled α-factor was performed as described previously (Toshima *et al.*, 2005). Briefly, cells were grown to an OD_600_ of ~0.3 in 50 ml YPD, briefly centrifuged and resuspended in 4 ml YPD containing 1% (w/v) BSA, 50 mM KH_2_PO_4_, pH 6, and 20 μg/ml of uracil, adenine, and histidine. After adding ^35^S-labeled α-factor, cell aliquots were withdrawn at various time points and subjected to a wash in pH 1.1 buffer to remove surface-bound α-factor so that internalized α-factor could be measured, or in pH 6 buffer to determine the total amount (internalized and bound) of α-factor. The amount of cell-associated radioactivity after each wash was determined by scintillation counting. Each experiment was performed at least three times. For the binding assay, cells grown to early to mid-logarithmic phase were briefly centrifuged, and resuspended in 50 μl SM with 1% (w/v) BSA and radiolabeled α-factor. After incubation on ice for 2 h, cells were washed with ice-cold SM and measured for their radioactivity.

### Nanoluc luciferase-based secretion assay

Nanoluc (Nluc) luciferase reporter plasmid was expressed as follows: the Nluc fragment that contains signal sequence of MFA1 (nt 1-267) was amplified by PCR using pNL1.1 (Promega, Madison, WI) as a template, and subcloned into the HindIII- and XhoI-digested pBlueScript II SK (pBS-Nluc), and the SacI-HindIII fragment of the 417-bp 5' UTR of *TPI1* gene, which contains the *S. cerevisiae TPI1* promoter, was amplified by PCR using yeast genome DNA as a template, and inserted into SacI- and HindIII-digested pBS-Nluc (pBS-P_*TPI*_1-Nluc). Next, the XhoI-ApaI fragment of the 200-bp 3' UTR of *TPI1* gene, which contains the *S. cerevisiae TPI1* terminator, was inserted into XhoI- and ApaI-digested pBS-P*TPI*1-Nluc (pBS-P_*TPI*_-Nluc-T_*TPI*_). To create an integration plasmid, the SacI-ApaI fragment of the P_*TPI*_-Nluc-T_*TPI*_ was inserted into the SacI- and ApaI-digested pRS305 (pRS305-P_*TPI*_-Nluc-T_*TPI*_). To integrate pRS305-P_*TPI*_-Nluc-T_*TPI*_ at the LEU2 locus, the plasmid was linearized by EcoRI and transformed into wild-type or mutant cells. For the secretion assay, cells expressing Nluc luciferase reporter were grown to an OD_600_ of ~0.5 in 1.0 ml YPD, briefly centrifuged, and resuspended in fresh 800 μl YPD. At each time point, 100 μl of the culture medium was aliquoted and centrifuged, and luciferase activities in the supernatants were measured by Nano-Glo luciferase assay system (Promega, Madison, WI).

## Results

### PS is required for cell surface localization of the Ste2 receptor

**Fig. 1:**
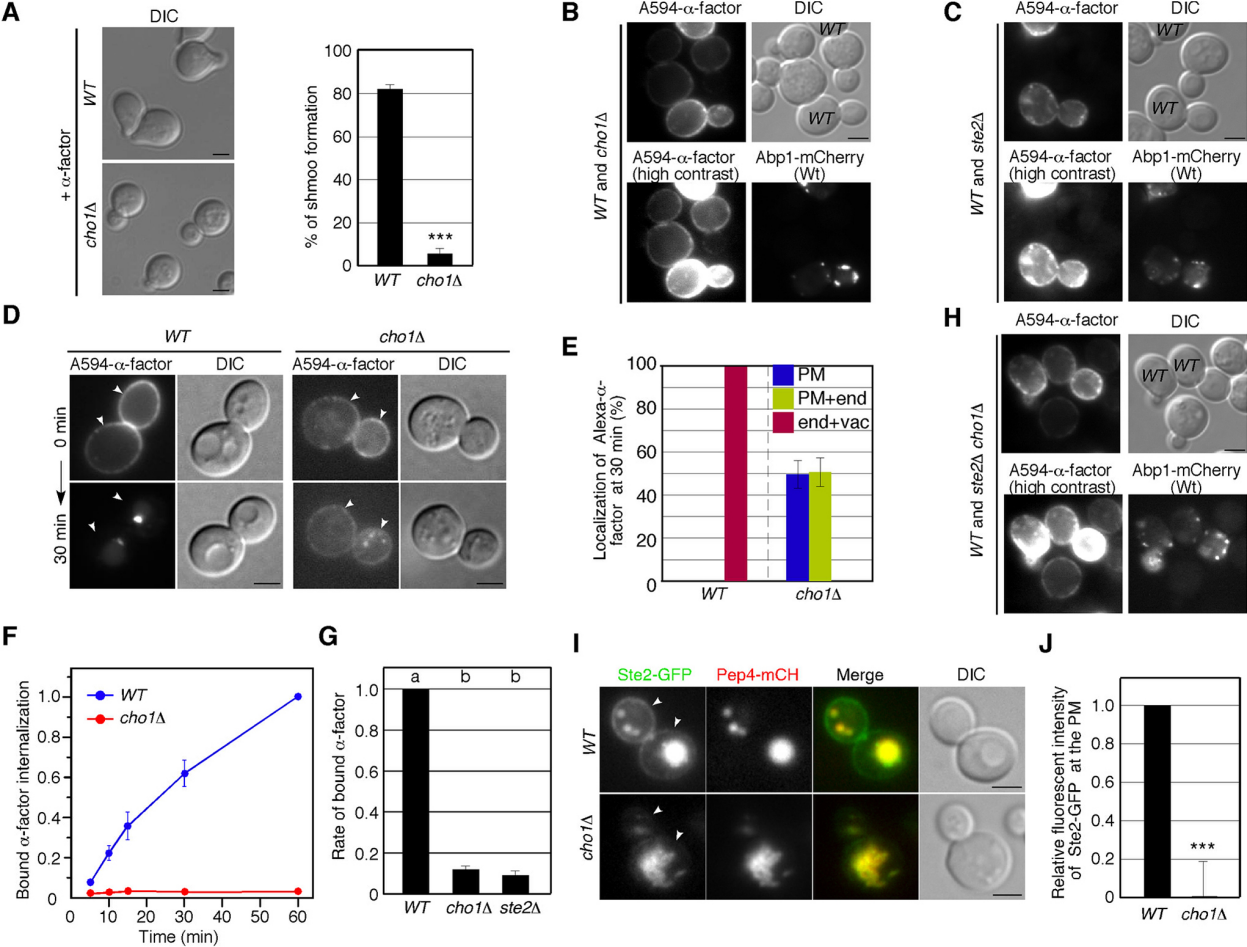
PS is required for the localization of Ste2 at the PM (A) Differential interference contrast (DIC) images of wild-type and *cho1*Δ cells grown to early logarithmic phase and treated with α-factor for 3 h. The bar graphs represent the percentages of cells exhibiting shmoo morphology. Data show mean ± standard deviation (SD) from three experiments. (B, C) Binding of A594-α-factor to wild-type and *cho1*Δ cells (B) or wild-type and *ste2*Δ cells (C). Cells were incubated with A594-α-factor on ice for 2 h, washed with ice-cold SM, and observed by fluorescence microscopy and DIC. (D) Wild-type and *cho1*Δ cells were treated with A594-α-factor, and the images were acquired 30 min after washing out unbound A594-α-factor and warming the cell to 25°C. Arrowheads indicate localization of A594-α-factor at the PM. (E) Quantification of localization of A594-α-factor in wild-type and *cho1*Δ cells. The bar graphs represent the percentages of cells exhibiting A594-α-factor localized at PM only (blue), PM and endosome (yellow), or endosome and vacuole (red) at 30 min after internalization. Data show mean ± standard deviation (SD) from three experiments, with 50 cells counted for each strain per experiment. (F) Radiolabeled α-factor internalization assays performed on the indicated strains at 25°C. Each curve represents the average of three independent experiments, and error bars indicate the SD at each time point. Data are shown as relative value of internalized amount of α-factor in wild-type cell at 60 min. (G) Relative amount of α-factor bound to wild-type, *cho1*Δ or *ste2*Δ cells. Error bars represent the SD from at least three experiments. Different letters indicate significant differences at *p*<0.005 between the cells (i.e., no significant difference for a vs. a, significant difference for a vs. b with *p*<0.005), one-way ANOVA with Tukey’s post-hoc test. Error bars indicate the standard SD from three experiments. (H) Binding of A594-α-factor to wild-type and *ste2*Δ *cho1*Δ cells. Cells were observed as described in (B, C). (I) Localizations of Ste2-GFP and Pep4-mCherry in wild-type and *cho1*Δ cell. Cells expressing Ste2-GFP and Pep4-mCherry were grown to early logarithmic phase in YPD medium at 25°C and observed by fluorescence microscopy and DIC. Arrowheads indicate localization of Ste2-GFP at the PM. (J) Quantification of relative fluorescent intensity of Ste2-GFP at the PM (*n* = 50 cells for each strain). ***, *p* value <0.005, unpaired *t*-test with Welch’s correction. Scale bars, 2.5 μm.

A previous study has demonstrated that PS is required for proper Cdc42 localization and establishment of cell polarity, and that PS depletion causes depolarization of Cdc42p, leading to a delay in bud emergence and, subsequently, defective mating (Fairn *et al.*, 2011). First, therefore, we confirmed that whether PS synthesis is required for the morphological change (shmoo morphology) in response to the mating pheromone α-factor. At 2 h after α-factor treatment, ~82% of wild-type cell exhibited shmoo morphology, whereas *cho1*Δ cells rarely did so (~4%) (Fig. 1A). To examine whether *cho1*Δ cells have defects in α-factor binding or in α-factor-induced signal transduction, we next examined the binding and uptake of α-factor into the cells. We labeled wild-type and *cho1*Δ cells with Alexa Fluor 594-conjugated α-factor (A594-α-factor) and observed the localization. We found that the fluorescence intensity of A594-α-factor bound to *cho1*Δ cells was decreased relative to wild-type cells (Fig. 1B). In contrast, A594-α-factor binding was barely observed in cells lacking the α-factor receptor Ste2p (Fig. 1C), confirming that α-factor only marginally binds to *cho1*Δ cells (Fig. 1C). We also observed an appreciable delay of A594-α-factor internalization in *cho1*Δ cells (Fig. 1D). Quantitative analysis categorizing A594-α-factor localization as PM only, PM and endosome, or endosome and vacuole revealed that *cho1*Δ cells had obvious defects in α-factor internalization (Fig. 1E). To examine in detail the effect of PS depletion on α-factor binding and uptake into the cell, we compared them using ^35^S-labeled α-factor. Similarly to A594-α-factor, ^35^S-labeled α-factor was efficiently incorporated into wild-type cells, whereas it was incorporated only slightly into *cho1*Δ cells (Fig. 1F). Surprisingly, binding of α-factor to *cho1*Δ cells was decreased to ~10% in comparison to wild-type cells, and the amount of binding was almost the same as that to *ste2*Δ cells (Fig. 1G). As A594-α-factor slightly bound to *cho1*Δ cells while ^35^S-labeled α-factor little bound, we speculated that changes in lipid composition in the PM by PS depletion might cause non-specific binding of A594-α-factor to the cell. As expected, we found that A594-α-factor barely binds to *ste2*Δ cells but binds to *ste2*Δ cells lacking Cho1p at the similar level to *cho1*Δ cells (Fig. 1B, H), suggesting that A594-α-factor binds to *cho1*Δ cells independently of Ste2 receptor. Additionally, we found that in *cho1*Δ cells Ste2p localization was markedly shifted to intracellular compartments, in contrast to its association with the PM in wild-type cells (Fig. 1I). While most wild-type cells obviously exhibited Ste2-GFP at the PM as well as the vacuole, *cho1*Δ cells showed little PM localization of Ste2p (Fig. 1I, J). Intracellular Ste2-GFP was highly colocalized with mCherry-fused Pep4p in both wild-type and *cho1*Δ cells (Fig. 1I), indicating that PS depletion leads to mis-sorting of Ste2p from the PM to the vacuole.

### PS depletion does not affect endocytic vesicle formation and internalization

**Fig. 2:**
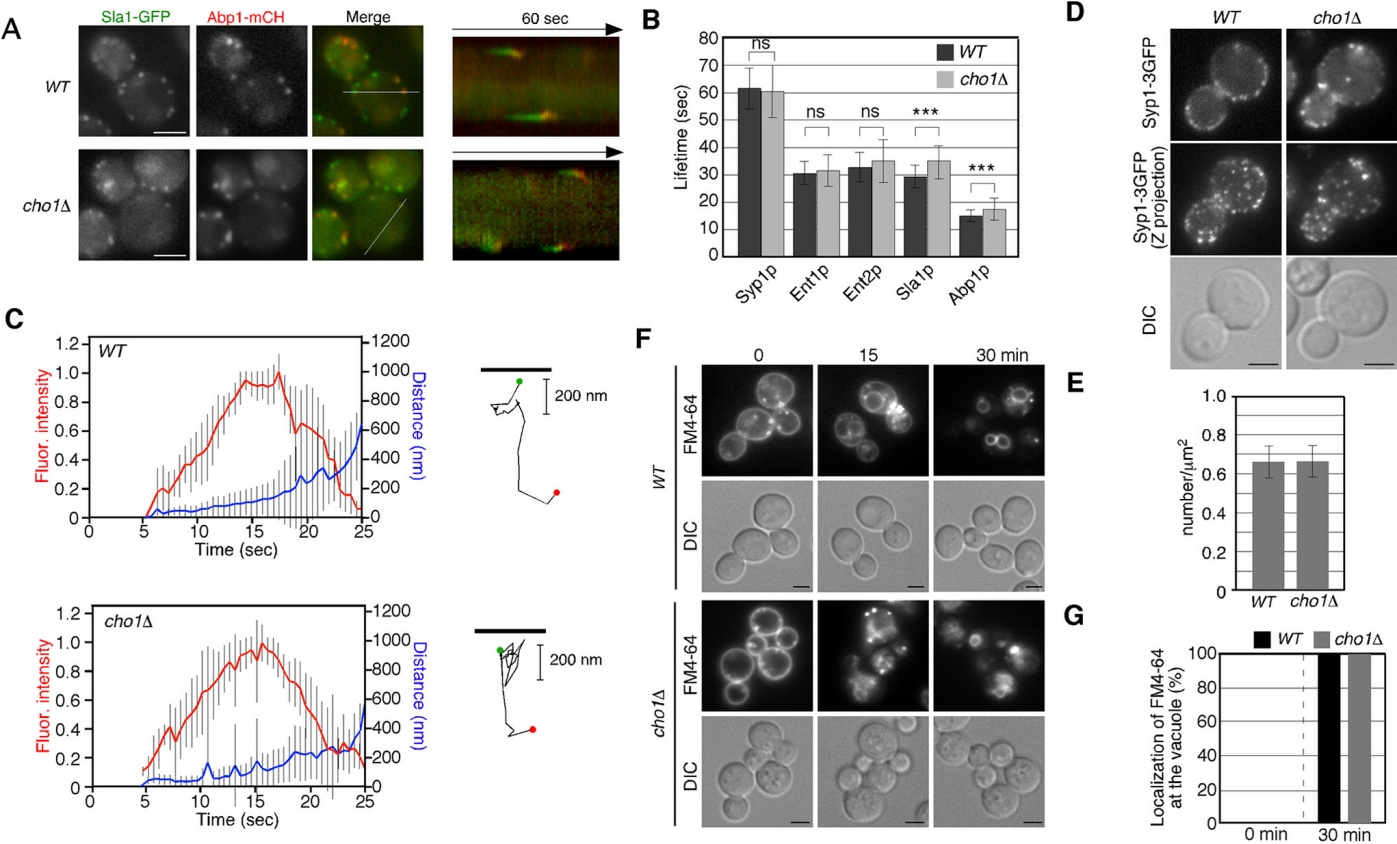
Effect of PS depletion on endocytic vesicle formation and internalization (A) Localizations of Sla1-GFP and Abp1-mCherry in wild-type and *cho1*Δ cell. Kymograph along lines in the merged images are shown in the right panels. (B) Average lifetimes of endocytic proteins ± SD for indicated strains. *n* = 50 patches for each strain. ***, *p* value <0.005, unpaired *t*-test with Welch’s correction. (C) Quantification of fluorescence intensity (red) and distance from the site of patch formation (blue) as a function of time for patches of Abp1-mCherry. Data from ten patches from each strain were averaged using single-color movies of Abp1-mCherry. Fluorescent intensity over time was corrected for photobleaching. Tracking of individual cortical Abp1p patches is indicated in right. Abp1-mCherry was visualized every 1 sec and patch movement traces were obtained for the entire life of the patches. Green and red dots indicate the first and the last position, respectively. (D) Localizations of Syp1-3GFP in wild-type and *cho1*Δ cell. The middle images show maximum intensity projections of Z stacks of wild-type and *cho1*Δ cells labeled with Syp1-3GFP. The Z series was acquired through the entire cell at 0.2-μm intervals. (E) Quantification of Syp1-3GFP/μm^2^ ± SD in wild-type and *cho1*Δ cells (*n* = 50 cells for each strain). (F) Bulk endocytosis in wild-type and *cho1*Δ cells. Cells were labeled with 200 μM FM4-64 for 15 min on ice. The images were acquired at 30 min after washing out unbound FM4-64 and warming the cell to 25°C. (G) The bar graph represents the percentages of cells exhibiting FM4-64 localized at the vacuole at 30 min after internalization. Data show the mean from at least three experiments, with 50 cells counted for each strain per experiment. Scale bars, 2.5 μm.

Given the reduced binding and internalization of fluorescent α-factor to the PM, we next investigated the effect of *CHO1* gene deletion on the formation and internalization of endocytic vesicles. We used Sla1-GFP and Abp1-mRFP as markers to follow the dynamics of endocytic vesicles (Fig. 2A) (Kaksonen *et al.*, 2003). First, we carried out simultaneous imaging of Sla1p and Abp1p to analyze the dynamic behavior of these proteins in live cells. In wild-type cells, Sla1-GFP was immediately followed by Abp1-mRFP recruitment, as shown in kymographs generated across a single pixel-wide line for an individual patch (Fig. 2A). Similar dynamics of Sla1-GFP and Abp1-mRFP patches were observed in *cho1*Δ cells (Fig. 2A), although the lifetime of Sla1-GFP was slightly prolonged in *cho1*Δ cells relative to wild-type cells (~29.4 vs. ~35.1 sec). This result is consistent with previous observations (Sun and Drubin, 2012). We next examined the effect of *CHO1* gene deletion on the dynamics of endocytic vesicles. Fluorescence intensity and particle tracking analysis showed that Abp1p patches in both wild-type and *cho1*Δ cells were internalized normally (Fig. 2C), although the lifetime of Abp1-mCherry in the *cho1*Δ cells was also slightly prolonged in comparison with that in wild-type cells (~15.1 vs. ~18.4 sec) (Fig. 2B). In contrast, the lifetimes of the early coat protein, Syp1p, and the mid coat proteins, Ent1p/2p, were almost the same in both wild-type and *cho1*Δ cells (Fig. 2B). To further examine the effect of PS depletion on clathrin coat assembly, we assessed the number of endocytic sites in *cho1*Δ cells using Syp1-3GFP (Suzuki *et al.*, 2012). Maximum-intensity Z-projections of live cells were analyzed to determine the average number of patches per unit surface area, and patch densities were quantified only in mother cells, where individual patches were distinguishable. As shown in Fig. 2D, in *cho1*Δ cells, there was no change in the number of Syp1p patches per unit surface area relative to wild-type cells. These results clearly demonstrated that endocytic vesicle formation and internalization occur normally in *cho1*Δ cells. Finally, we examined the internalization of 3-triethylammoniumpropyl-4-p-diethylaminophenylhexatrienyl pyridinium dibromide (FM4-64), a lipophilic styryl dye that is used to label bulk endocytosis. When added to wild-type cells, FM4-64 is immediately incorporated into the PM, internalized via bulk endocytosis, and then transported to the vacuole within 30 min (Fig. 2F, G). This assay revealed that *cho1*Δ cells showed little delay in membrane internalization, suggesting that the endocytic machinery is not impaired in the *cho1*Δ cell.

### PS is required for the localization of cell surface proteins at the PM

**Fig. 3:**
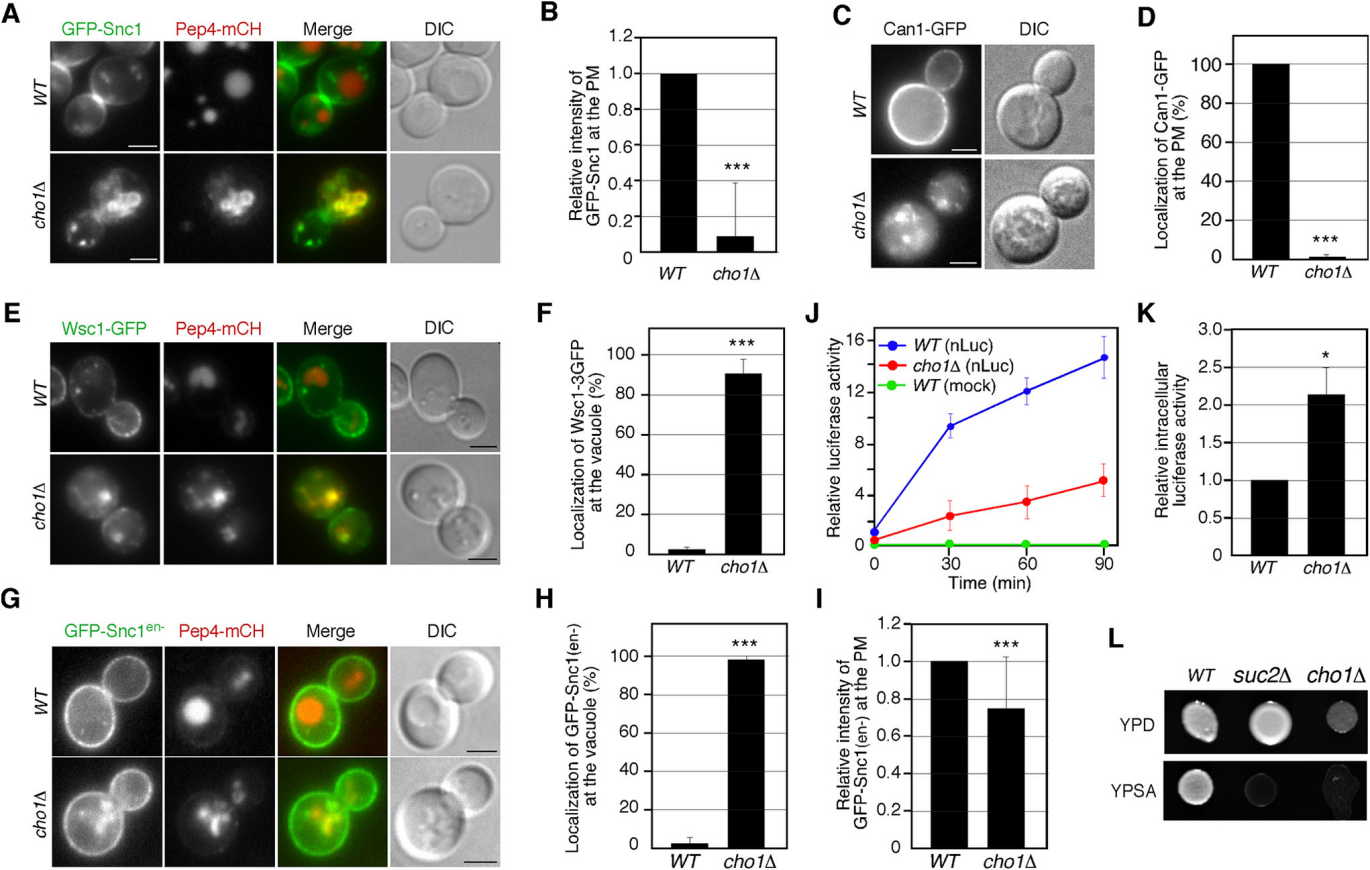
PS is required for recycling of cell surface proteins to the PM (A) Localization of GFP-Snc1 and Pep4-mCherry in wild-type or *cho1*Δ cells. Cells expressing GFP-Snc1 and Pep4-mCherry were grown to early logarithmic phase in YPD medium at 25°C and observed by fluorescence microscopy and DIC. (B) Quantification of relative fluorescent intensities of GFP-Snc1 at the PM. (*n* = 60 cells for each strain). (C) Localization of Can1-GFP in wild-type and *cho1*Δ cells. Cells expressing Can1-GFP were grown to early logarithmic phase in synthetic medium supplemented with 2% glucose and low levels of amino acids at 25°C. (D) The bar graph represents the percentages of cells exhibiting Can1-GFP localization at the PM. Data show mean ± SD from three experiments, with 50 cells counted for each strain per experiment. (E) Localization of Wsc1-GFP and Pep4-mCherry in wild-type or *cho1*Δ cells. Cells expressing GFP-Snc1 and Pep4-mCherry were grown and observed, as described in (A). (F) The bar graph represents the percentages of cells exhibiting Wsc1-GFP localization at the vacuole. Data show mean ± SD from three experiments, with 50 cells counted for each strain per experiment. (G) Localization of GFP-Snc1(en-) and Pep4-mCherry in wild-type and *cho1*Δ cells. Cells expressing GFP-Snc1(en-) were grown and observed, as described in (A). (H) The bar graph represents the percentages of cells exhibiting GFP-Snc1(en-) localization at the vacuole. Data show mean ± SD from three experiments, with 50 cells counted for each strain per experiment. (I) Quantification of relative fluorescent intensities of GFP-Snc1(en-) at the PM. (*n* = 50 cells for each strain). (J) NanoLuc luciferase-based secretion assays performed on the indicated strains. Each curve represents the average of three independent experiments, and error bars indicate the SD at each time point. Data are shown as relative value of luciferase activity in culture media of wild-type cell at 0 min. (K) The bar graph represents intracellular luciferase activities in wild-type and *cho1*Δ cells. Data show mean ± SD from four experiments. (L) Plates showing the growth phenotype of wild-type and *cho1*Δ cells. Cells were plated onto YPD or yeast pepton sugar agar (YPSA) medium containing sucrose as a sole carbon source at 25°C. ***, *p* value <0.005, unpaired *t*-test with Welch’s correction. Scale bars, 2.5 μm.

To clarify whether the decreased localization of Ste2p at the PM in *cho1*Δ cells is caused by a recycling defect, we examined the effect of *cho1*Δ on trafficking of GFP-Snc1p, an exocytic v-SNARE that is endocytosed, transiently localized to early endosomes, and recycled back to the PM via the *trans-*Golgi network (TGN) (Galan *et al.*, 2001; Lewis *et al.*, 2000). It has been shown that mutations affecting endosome-mediated trafficking often cause mis-localization of Snc1p from the PM to the endosomal or vacuolar compartments (Galan *et al.*, 2001; Lewis *et al.*, 2000; Reggiori *et al.*, 2003). In wild-type cells, GFP-Snc1p was localized at the PM with some punctate staining of internal structures (Fig. 3A), as shown in previous studies (Lewis *et al.*, 2000). Intriguingly, in *cho1*Δ cells, localization of GFP-Snc1 was shifted to intracellular compartments (Fig. 3A). Quantitative analysis showed that the fluorescence intensity of GFP-Snc1 at the PM was decreased to ~9% in *cho1*Δ cells relative to that in wild-type cells (Fig. 3B). These intracellular structures were significantly co-localized with Pep4-mCherry, a marker for the vacuole (Fig. 3A). To examine whether PS synthesis is generally required for the localization of cell surface protein at the PM, we used the yeast arginine permease Can1p, which is known to be recycled to the PM via the TGN after endocytic internalization (Gournas *et al.*, 2017). Similarly to Ste2p, most wild-type cells exhibited localization of Can1-GFP at the PM, but the cell surface localization was markedly decreased to ~1.3% in *cho1*Δ cells (Fig. 3C, D). We also examined the localization of the cell wall sensor protein Wsc1p, which is maintained by endocytosis and recycling from endosomes back to the cell surface (Piao *et al.*, 2007; Ueno *et al.*, 2014). While GFP-tagged Wsc1p is localized primarily at the PM in wild-type cells, it was localized at the vacuole in *cho1*Δ cells (Fig. 3E), suggesting that PS is generally required for the localization of cell surface protein at the PM. Quantitative analysis showed that only ~2.7% of wild-type cells had Wsc1-GFP at the vacuole, whereas ~90.7% of *cho1*Δ cells exhibited vacuolar localization (Fig. 3F). To further characterize the GFP-Snc1 localization defect in *cho1*Δ cells, we used a GFP-fused Snc1(en-) mutant containing two mutations, V40A and M43A, that interfere with endocytosis and cause GFP-Snc1 to accumulate on the PM in wild-type cells (Fig. 3G) (Lewis *et al.*, 2000). We observed that GFP-Snc1(en-) was localized to the vacuole in addition to the PM in *cho1*Δ cells, suggesting defective transport from the TGN to the PM (Fig. 3G, H). Consistent with this, the fluorescence intensity of GFP-Snc1(en-) at the PM was decreased to ~75.6% in *cho1*Δ cells relative to that in wild-type cells (Fig. 3I). To further confirm this, we examined the effect on the secretory pathway using NanoLuc (Nluc) luciferase reporter, which contains a secretory signal for α-factor at the N terminus. The Nluc luciferase reporter gene was chromosomally integrated in both wild-type and *cho1*Δ cells, and the luciferase activity in the culture medium was assessed in both cases. Little luciferase activity was detected in the culture supernatant of wild-type cells, which did not express Nluc luciferase (mock), whereas the activity increased steadily until the end of measurement in cells expressing Nluc luciferase (Fig. 3J). Interestingly, the luciferase activity in the culture supernatant of *cho1*Δ cells was significantly decreased while the intracellular activity was increased (Fig. 3J, K). Additionally, we examined the growth of *cho1*Δ cells on media containing sucrose as a sole carbon source, because secretion of invertase, encoded by *SUC2*, is prerequisite for yeast cell growth on the media (Fig. 3L) (Novick *et al.*, 1980). Consistent with the result obtained using the NLuc luciferase reporter, the *cho1*Δ cells were unable to grow on media containing sucrose (Fig. 3L). These results, taken together, suggested that *cho1*Δ cells have a defect at least in the pathway from the TGN to the PM, and that cell surface proteins recycled back to the PM might be mis-sorted to the vacuole at the TGN.

### PS sensors accumulate at the intracellular compartments in yeast mutants with a defective recycling pathway

**Fig. 4:**
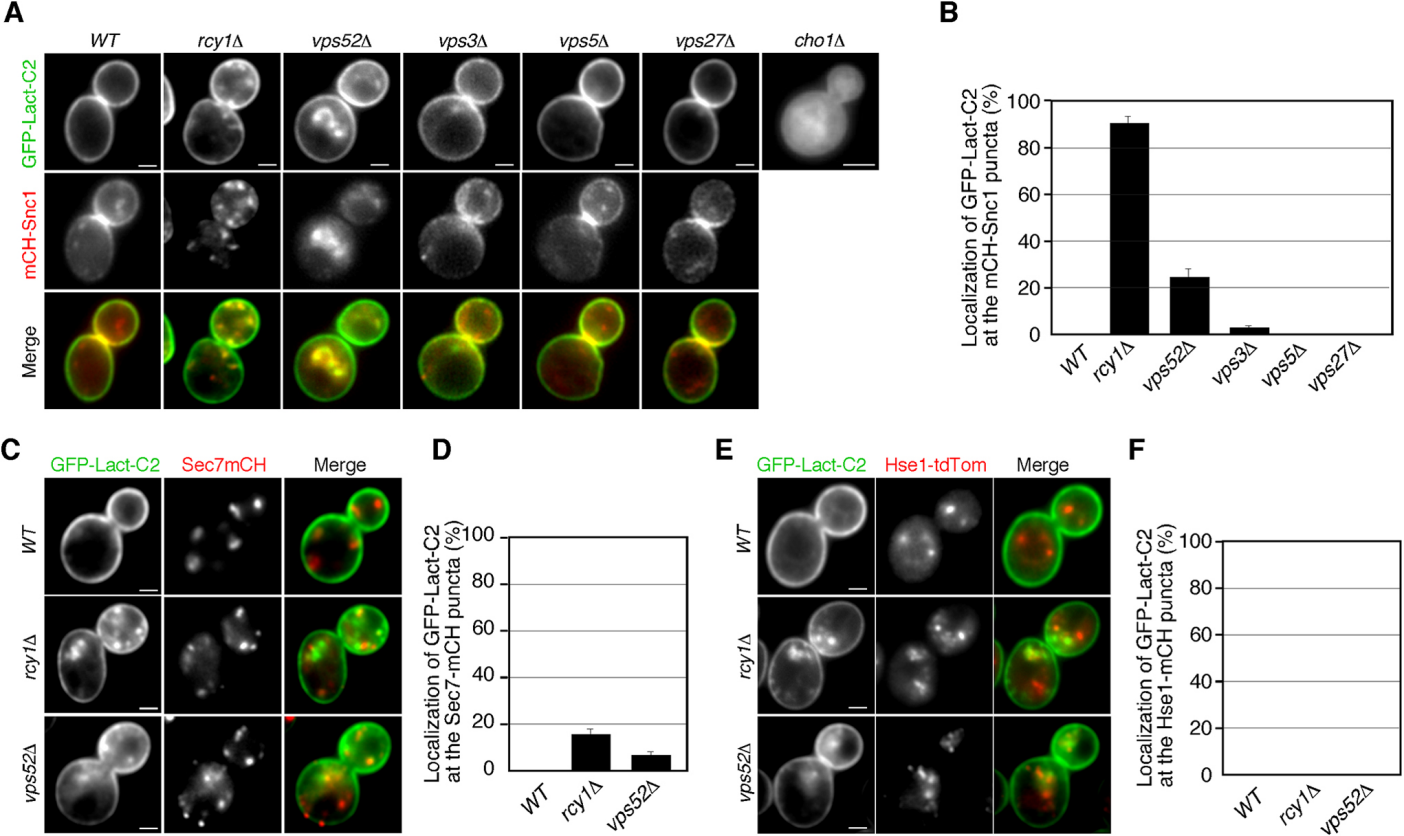
Localization of PS in yeast mutants with a defective endocytic-recycling pathway (A) Localization of Lact-C2-GFP and mCherry-Snc1 in wild-type and indicated mutant cells. Cells expressing Lact-C2-GFP and mCherry-Snc1 were grown to early logarithmic phase in YPD medium at 25°C and observed by fluorescence microscopy. (B) Quantification of colocalization of Lact-C2-GFP and mCherry-Snc1 at the intracellular compartments in wild-type and indicated mutant cells. Error bars indicate the SD from at least three independent experiments. The percentages of colocalization were calculated as the ratio of Lact-C2-GFP localized in intracellular mCherry-Snc1 puncta (*n* = 100) in each experiment. (C, D) Localization of Lact-C2-GFP and Sec7-mCherry (C) or Hse1-tdTomato (D) in wild-type or indicated mutant cells. Cells were grown and observed, as described in (A). (E, F) Quantification of colocalization of Lact-C2-GFP and Sec7-mCherry (D) or Hse1-tdTomato in wild-type and indicated mutant cells. Error bars indicate the standard deviation from at least three independent experiments. The percentages of colocalization were calculated as the ratio of Lact-C2-GFP localized in Sec7-mCherry or Hse1-tdTomato puncta (*n* = 100). Scale bars, 2.5 μm.

To evaluate the potential function of PS in cell surface protein recycling, we next investigated its subcellular localization using the stereospecific PS-binding C2 domain of bovine lactadherin (Lact-C2) (Leventis and Grinstein, 2010; Yeung *et al.*, 2008). First, we introduced a fluorescent PS-specific probe, GFP-fused Lact-C2 (GFP-Lact-C2), to wild-type and *cho1*Δ cells and observed its localization. As shown previously, in wild-type cells, Lact-C2-GFP was localized exclusively at the PM, whereas it was largely cytoplasmic in *cho1*Δ cells (Fig. 4A) (Fairn *et al.*, 2011; Sun and Drubin, 2012). As described above, as *cho1*Δ cells have severe defects in the TGN-to-PM pathway but show normal endocytic internalization, we speculated that PS might transiently localize at intracellular compartments, such as the TGN and endosomes, and regulate the recycling pathway. Previous studies have shown that in cells lacking Rcy1p, an F-box protein involved in the recycling of cell surface proteins, Lact-C2-GFP accumulates at intracellular membrane compartments and co-localizes with mRFP-Snc1 there (Galan *et al.*, 2001; Ma and Burd, 2020; Wiederkehr *et al.*, 2000). As reported previously, we observed that GFP-Lact-C2 was highly co-localized with mCherry-Snc1p at intracellular puncta in *rcy1*Δ cells (~90.0%) (Fig. 4B, C). Previous studies have also demonstrated that in *rcy1*Δ cells Snc1p partially co-localizes with Tlg1p at the early endosome, but does not co-localize with Sec7p, which is localized at the TGN (Best *et al.*, 2020; Ma and Burd, 2019). Similarly to the case of Snc1p and Sec7p, GFP-Lact-C2 was not well co-localized with Sec7p-residing TGN (~14.7%) in the *rcy1*Δ mutant (Fig. 4C, D). We also compared the PS localization with Hse1-tdTomato, a marker of endosomal compartments, but no co-localization was observed (Fig. 4E, F). These results suggest that neither the TGN nor endosome is the region that PS accumulates. To further examine possible intracellular compartments at which PS is localized, we utilized three other mutants with defects in the intracellular vesicle trafficking pathway. Among them, we found that Lact-C2-GFP also accumulates at intracellular membrane compartments in *vps52*Δ cells. The *VPS52* gene encodes a subunit of the GARP (Golgi-associated retrograde protein) complex, which is a protein complex involved in the recycling of proteins from endosomes to the TGN (Bonifacino and Hierro, 2011). In about 24% of the *vps52*Δ mutants, GFP-Lact-C2 was localized at intracellular puncta where Snc1p resides, but Sec7p and Hse1p do not (Fig. 4B–F). This indicated that transport of PS from the endosome to the TGN is partially impaired in the *vps52*Δ mutant. In contrast, GFP-Lact-C2 exhibited PM localization, similar to that in wild-type cells, in cells lacking the *VPS3*, *VPS5* or *VPS27* gene, which encode proteins required for the early-to-late endosome, late endosome-to-TGN, or late endosome-to-vacuole trafficking (Fig. 4A) (Bowers and Stevens, 2005). These observations suggest a possibility that PS is endocytosed and recycled back to the PM through the endosome and the TGN compartments.

### PS depletion causes an increase in the number of endosomal compartments

**Fig. 5:**
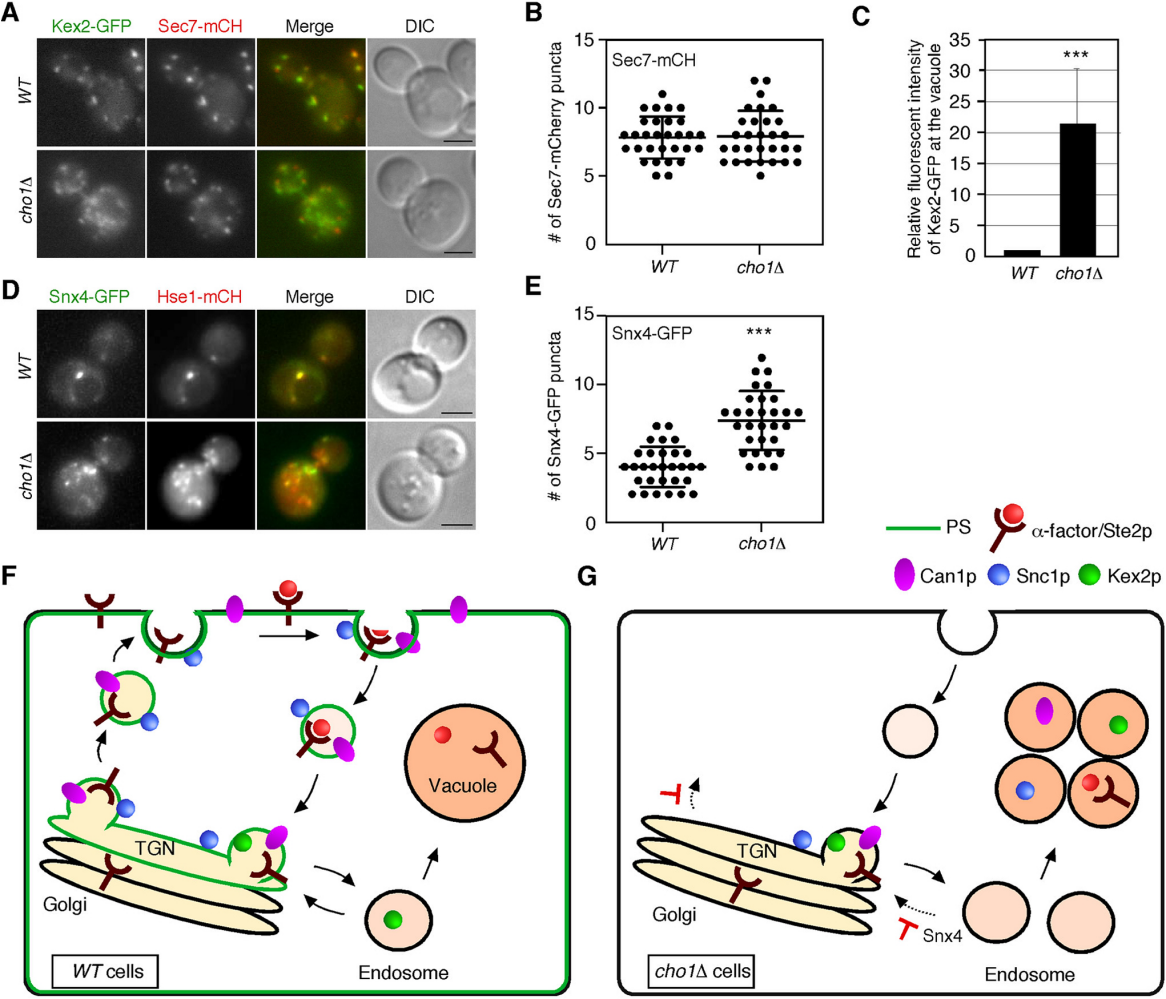
PS depletion causes an increase in the number of Snx4p-residing endosomes (A) Localization of Kex2-GFP and Sec7-mCherry in wild-type and *cho1*Δ cells. Cells expressing Kex2-GFP and Sec7-mCherry were grown to early logarithmic phase in YPD medium at 25°C and observed by fluorescence microscopy. (B) Quantification of the number of Sec7p-residing TGN displayed in (A). Data show mean ± SD (*n* = 30 cells for each strain). (C) Quantification of the fluorescence intensity of Kex2-GFP at the vacuole. Quantification of relative fluorescent intensity of Ste2-GFP at the PM (*n* = 50 cells for each strain). (D) Localization of Snx4-GFP and Hse1-tdTomato in wild-type and *cho1*Δ cells. Cells were grown and observed, described in (A). (E) Quantification of the number of Snx4p-residing endosome displayed in (D). Data show mean ± SD (*n* = 30 cells for each strain). ***, *p* value <0.005, unpaired *t*-test with Welch’s correction. Scale bars, 2.5 μm. (F, G) Model for the localization and function of PS. PS localizes at the PM and intracellular compartments in the recycling pathway in wild-type cell (F). In *cho1*Δ cell, transport from the endosome to the TGN is suppressed, resulting in mis-sorting of Ste2p, Can1p and Kex2 to the vacuole. The *cho1*Δ cell also has defects in vesicle transport from the TGN to the PM. See details in the text. Red T bars indicate the steps that show defects in the absence of Cho1p.

Since PS appears to localize transiently at the TGN and endosome, we next examined the effects of PS depletion on these compartments. First, we examined the localization of Kex2, which is known to localize to both the TGN and endosomal compartments. Kex2p is a pheromone-processing protease residing at the TGN, being transported to the endosome and then recycled back to the TGN in a retromer-dependent manner (Lewis *et al.*, 2000; Seaman *et al.*, 1998). In *cho1*Δ cells the number of Sec7p-residing TGN was almost the same as that in wild-type cells (Fig. 5A, B), but the fluorescence intensity of Kex2-GFP at the vacuole was significantly increased (Fig. 5C). These observations indicated that the retrieval pathway from the endosome to the TGN is impaired in *cho1*Δ cells. Next, to determine the effect on endosomes in *cho1*Δ cells, we examined the localization of Snx4p, which is a subunit of the sorting nexin complex that mediates a retrieval pathway from the endosome to the TGN (Hettema *et al.*, 2003). GFP-Snx4 puncta were well co-localized with Hse1-tdTomato-residing endosomes in both wild-type and *cho1*Δ cells (Fig. 5D). Intriguingly, we found that the number of these GFP-Snx4-residing puncta was significantly increased in *cho1*Δ cells (Fig. 5D, E). These observations suggest that PS depletion affects transport from the endosome to the TGN, resulting in an increase in the number of endosomal compartments.

## Discussion

In this study, we have shown that PS synthesis is important for the localization of cell surface proteins. A previous study had demonstrated that cells lacking *CHO1* gene have significantly decreased mating efficiency due to impairment of the polarity of Cdc42p, which is required for the formation of mating projections (Fairn *et al.*, 2011). In the study it had also shown that PS depletion in *cho1*Δ cell causes a decrease in Phosphatidylehanolamine (PE) content, but Cdc42 accumulation correlates with the presence and polarization of PS, and not changes in PE content (Fairn *et al.*, 2011). In the present study, we demonstrated that *cho1*Δ cells have defect in the localization of cell surface proteins at the PM. Thus, it seems that the decreased mating efficiency observed in *cho1*Δ cells is caused by defective transport of Ste2p from the TGN to the PM (Fig. 5F, G). While it is known that binding to α-factor triggers Ste2p transport through the endocytic pathway to the vacuole (Hicke and Riezman, 1996; Toshima *et al.*, 2009), it is still unclear whether Ste2p in the absence of α-factor is recycled back to the PM. In our previous study, we showed that cells lacking the Arf-GAP Glo3p exhibit a dramatic reduction of α-factor binding to the cell surface (Kawada *et al.*, 2015). The *glo3*Δ mutant had prominent defects in endosome-to-TGN trafficking, suggesting that Ste2p cannot be efficiently recycled back to the PM (Kawada *et al.*, 2015). In *cho1*Δ cells, several cell surface proteins, including Ste2p, were mis-transported to the vacuole. We also showed that the GFP-Snc1(en-) mutant, which is not endocytosed, was localized to both the PM and the vacuole, indicating that TGN-to-PM transport was not completely disrupted. These observations suggest that defects in both the secretion and recycling pathways would decrease the localization of Ste2p at the PM in *cho1*Δ cells (Fig. 5G). In wild-type cells, we propose that Ste2p is also endocytosed constitutively, but then retrieved to the TGN from the endosomal compartment and shuttled again back to the cell surface (Fig. 5F).

Several studies have reported that PS is required for intracellular vesicle transport and that *cho1*Δ cells exhibit defects in endocytic site formation (Sun and Drubin, 2012). It has also been shown that in *cho1*Δ cells Sla1p does not preferentially localize in the small buds where endocytic actin patches are normally concentrated (Sun and Drubin, 2012). Similarly to these previous observations, we have shown that most *cho1*Δ cells lose their polarized localization of endocytic sites. However, the localization, lifetime, and amount of Syp1p, which localizes at the earliest stage of endocytosis, appear to be mostly normal, suggesting that endocytic site formation in the mutant is not severely affected. Additionally, actin patch dynamics were almost equivalent in both wild-type and *cho1*Δ cells. Thus, it remains unclear why the lifetimes of Sla1p and Abp1p are prolonged in *cho1*Δ cells, and further studies to determine the role of PS in the endocytic pathway will be needed.

In mammalian cells, PS is known to be important for retrograde membrane traffic at recycling endosomes (Uchida *et al.*, 2011). PS localizes preferentially at recycling endosomes, and masking of intracellular PS by overexpression of Lact-C2 suppressed membrane traffic from REs to the Golgi (Uchida *et al.*, 2011). In the process of retrograde trafficking, a dynamin-like ATPase EHD1 has been shown to be recruited to endosomes through its PS-binding ability (Lee *et al.*, 2015). EHD1 facilitates the fission of tubulovesicular carriers from endosomes, and a reduction of PS decreases the localization of EHD1 at endosomes containing SNX1, a component of the SNX1-BAR complex, resulting in the fission defect (Deo *et al.*, 2018; Kamerkar *et al.*, 2019; Kawasaki *et al.*, 2022). In yeast, it has demonstrated that the SNX-BAR protein, Snx4-Atg20 dimer, also preferentially binds and remodels the PS-containing membrane, thereby exporting the PS-rich membrane from the endosome to promote autophagy and vacuole membrane fusion (Ma *et al.*, 2018). Additionally, a previous study reported that PS flipping to the cytosolic leaflet of endosomal membrane by yeast Drs2p, a phospholipid translocase in the type IV P-type ATPase family, is crucial for generating membrane curvature at the endosomes (Xu *et al.*, 2013). This Drs2p flippase activity is required for Gcs1 endosomal localization via its +ALPS motif and protein transport between early endosome and the TGN (Xu *et al.*, 2013), suggesting a role of PS in the vesicle transport between these organelles. Our present findings also suggest the general importance of PS in the recycling of cell surface proteins. In cells lacking Rcy1p or Vps52p, we have shown that PS accumulates at intracellular membrane compartments that mediate endosome-to-Golgi trafficking. In contrast, in cells lacking Vps3p, Vps5p, or Vps27p, PS localization appears to be normal. Vps3p is a subunit of the CORVET complex involved in early-to-late endosomal maturation (Nordmann *et al.*, 2010), and Vps5p, a subunit of the retromer complex, mediates transport from the prevacuolar endosomal compartment to the TGN (Nothwehr *et al.*, 2000; Seaman, 2007). Vps27p recruits the ESCRT machinery to endosomes during multivesicular body (MVB) sorting (Katzmann *et al.*, 2003). Deletion of these proteins is known to cause mis-sorting of cargos to abnormal endosome structures or vacuoles (Bowers and Stevens, 2005). Thus, the normal localization of Lact-C2 to the PM suggests that PS is localized at the early-stage endosome, and not transported to the late endosomal compartment. This idea is consistent with a previous observation that retrograde sorting of PS from the early endosome by Snx4 family sorting nexins is important for ensuring that vacuolar fusion occurs at a later stage (Ma *et al.*, 2018). Our finding that the number of endosomes containing Snx4p was increased in *cho1*Δ cells also supports this idea.

## Conflict of Interest

The authors declare that there are no conflicts of interest.
