# Supplementary material for: Role of phosphatidylserine in the localization of cell surface membrane proteins in yeast
Source: Cell Struct Funct. 2022 Dec 15;48(1):19–30. doi: 10.1247/csf.22081 (PMC10725852; doi:10.1247/csf.22081)
Supplement: Supplementary file 1 — Supplementary Table 1 [file csf_48_22081_1.zip › Table_S1.docx]

**Table S1. Yeast strains**

Strain Genotype Source

JJTY0132 *Mat*α *his3*-Δ*200* *leu2-3*, *112* *ura3-52 lys2-801 ENT1-GFP*::*HIS3* Toshima lab

JJTY0501 *Mat***a** *his3*Δ*1* *leu2*Δ*0 ura3*Δ*0* *lys2*Δ*0 bar1*Δ::*LEU2* Toshima lab

JJTY0525 *Mat***a** *his3*-Δ*200* *leu2-3*, *112* *ura3-52* *bar1*Δ::*LEU2 ste2Δ*::*KanMX6* Toshima lab

JJTY1219 *Mat***a** *his3*Δ*1* *leu2*Δ*0* *ura3*Δ*0 lys2*Δ*0* This study

JJTY1225 *Mat***a** *his3*Δ*1* *leu2*Δ*0* *ura3*Δ*0 lys2*Δ*0 SYP1-3GFP*::*HIS3* This study

JJTY3398 *Mat***a** *his3*Δ*1* *leu2*Δ*0* *ura3*Δ*0 met15*Δ*0 Ste2-GFP*::*HIS3 PEP4-mCherry*::*URA* This study

JJTY3492 *Mat***a** *his3*-Δ*200* *leu2-3*, *112* *ura3-52 lys2-801 SLA1-GFP*::*HIS3 ABP1-mCherry*::*URA3* This study

JJTY3668 *Mat***α** *his3*-Δ*200* *leu2-3*, *112* *ura3-52 lys2-801, cho1Δ::KanMX6* This study

JJTY3670 *Mat***a** *his3*-Δ*200* *leu2-3*, *112* *ura3-52 lys2-801, cho1Δ::KanMX6 bar1Δ::LEU2* This study

JJTY3669 *Mat***a** *his3*-Δ*200* *leu2-3*, *112* *ura3-52 lys2-801, cho1Δ::KanMX6 SLA1-GFP*::*HIS3*

*ABP1-mCherry*::*URA3* This study

JJTY3902 *Mat***a** *his3*-Δ*200* *leu2-3*, *112* *ura3-52 lys2-801 cho1*Δ::*KanMX6 SYP1-3GFP*::*HIS3* This study

JJTY3907 *Mat***a** *his3*-Δ*200* *leu2-3*, *112* *ura3-52 lys2-801 cho1*Δ::*KanMX6 ENT1-GFP*::*HIS3* This study

JJTY3910 *Mat***a** *his3*-Δ*200* *leu2-3*, *112* *ura3-52 lys2-801 cho1*Δ::*KanMX6 ENT2-GFP*::*HIS3* This study

JJTY3911 *Mat***a** *his3*-Δ*200* *leu2-3*, *112* *ura3-52 lys2-801 cho1*Δ::*KanMX6 STE2-GFP*::*HIS3* This study

JJTY3914 *Mat***a** *his3*-Δ*200* *leu2-3*, *112* *ura3-52 cho1*Δ::*KanMX6 CAN1GFP*::*HIS3* This study

JJTY4999  *Mat*α *his3*-Δ*200* *leu2-3*, *112* *ura3-52 lys2-801 PEP4-mCherry*::*URA3 SNC1-GFP*::*HIS* This study

JJTY5006 *Mat***α** *his3*-Δ*200* *leu2-3,112* *ura3-52* *lys2-801 WSC1-3GFP*::*HIS3 PEP4-mCherry*::*URA3* This study

JJTY5008 *Mat***α** *his3*-Δ*200* *leu2-3,112* *ura3-52* *lys2-801 cho1Δ*::*KanMX6 WSC1-3GFP*::*HIS3*

*PEP4-mCherry*::*URA3* This study

JJTY5029 *Mat***a** *his3*Δ*1* *leu2*Δ*0* *ura3*Δ*0 met15*Δ*0 cho1Δ::KanMX6 Ste2-GFP*::*HIS3 PEP4-mCherry* ::*URA* This study

JJTY5240 *Mat***α** *his3*Δ*1* *leu2*Δ*0* *ura3*Δ*0 ENT1-GFP*::*HIS3* This study

JJTY5241 *Mat***α** *his3*Δ*1* *leu2*Δ*0* *ura3*Δ*0 ENT2-GFP*::*HIS3* This study

JJTY6362 *Mat***a** *his3-Δ200 leu2-3, 112 ura3-52 lys2-801 GFP-SNX4*::*HIS3 HSE1-tdTomato*::*URA3* This study

JJTY6363 *Mat***a** *his3-Δ200 leu2-3, 112 ura3-52 lys2-801 cho1*Δ::*kanMX6 GFP-SNX4*::*HIS3*

*HSE1-tdTomato*::*URA3* This study

JJTY6404 *Mat***a** *his3-Δ200 leu2-3, 112 ura3-52 lys2-801 cho1*Δ::*kanMX6 STE2-GFP*::*HIS3*

*PEP4-mCherry*::*URA3* This study

JJTY0646 *Mat* **a** *his3*-Δ*200* *leu2*,*3-112* *ura3-52* *trp1-Δ901 lys2-801 suc2*Δ This study

JJTY6407 *Mat***a** *his3-Δ200 leu2-3, 112 ura3-52 lys2-801 cho1*Δ::*kanMX6 GFP-SNC1*::*HIS3*

*PEP4-mCherry*::*URA3* This study

JJTY6412 *Mat***a** *his3-Δ200 leu2-3, 112 ura3-52 lys2-801 GFP-snc1(en-)*::*HIS3 PEP4-mCherry*::*URA3* This study

JJTY6413 *Mat***a** *his3-Δ200 leu2-3, 112 ura3-52 lys2-801 cho1*Δ::*kanMX6 GFP-snc1(en-)*::*HIS3*

*PEP4-mCherry*::*URA3* This study

JTY11789 *Mat***a** *his3*-Δ*200* *leu2-3*, *112* *ura3-52* *bar1*Δ::*LEU2 ste2Δ*::*KanMX6 cho1Δ*:: *NatMX4* This study

JJC2006 *Mat***a** *his3*Δ*1* *leu2*Δ*0* *ura3*Δ*0* *lys2*Δ*0* GFP-Lact-C2::*HIS3* mCherry-Snc1::*URA3* This study

JJC2026 *Mat***a** *his3*Δ*1* *leu2*Δ*0* *ura3*Δ*0* *lys2*Δ*0* GFP-Lact-C2::*HIS3* mCherry-Snc1::*URA3 vps27*Δ::*KanMX* This study

JJC2036 *Mat***a** *his3*Δ*1* *leu2*Δ*0* *ura3*Δ*0* *lys2*Δ*0* GFP-Lact-C2::*HIS3* mCherry-Snc1::*URA3 vps3*Δ::*KanMX* This study

JJC2186 *Mat***a** *his3*Δ*1* *leu2*Δ*0* *ura3*Δ*0* *lys2*Δ*0* GFP-Lact-C2::*HIS3* mCherry-Snc1::*URA3 vps5*Δ::*KanMX* This study

JJC2142 *Mat***a** *his3*Δ*1* *leu2*Δ*0* *ura3*Δ*0* *lys2*Δ*0* GFP-Lact-C2::*HIS3* Hse1-tdTomato::*URA3 rcy1*Δ::*KanMX* This study

JJC2144 *Mat***a** *his3*Δ*1* *leu2*Δ*0* *ura3*Δ*0* *lys2*Δ*0* GFP-Lact-C2::*HIS3* Sec7-mCherry::*URA3 rcy1*Δ::*KanMX* This study

JJC2146 *Mat***a** *his3*Δ*1* *leu2*Δ*0* *ura3*Δ*0* *lys2*Δ*0* GFP-Lact-C2::*HIS3* mCherry-Snc1::*URA3 rcy1*Δ::*KanMX* This study

JJC2092 *Mat***a** *his3*Δ*1* *leu2*Δ*0* *ura3*Δ*0* *lys2*Δ*0* GFP-Lact-C2::*HIS3* Hse1-tdTomato::*URA3 vps52*Δ::*KanMX* This study

JJC2094 *Mat***a** *his3*Δ*1* *leu2*Δ*0* *ura3*Δ*0* *lys2*Δ*0* GFP-Lact-C2::*HIS3* Sec7-mCherry::*URA3 vps52*Δ::*KanMX* This study

JJC2096 *Mat***a** *his3*Δ*1* *leu2*Δ*0* *ura3*Δ*0* *lys2*Δ*0* GFP-Lact-C2::*HIS3* mCherry-Snc1::*URA3 vps52*Δ::*KanMX* This study
